# Copper(i) as a reducing agent for the synthesis of bimetallic PtCu catalytic nanoparticles†

**DOI:** 10.1039/d3na00158j

**Published:** 2023-07-20

**Authors:** Adrián Fernández-Lodeiro, Javier Fernández Lodeiro, Noelia Losada-Garcia, Silvia Nuti, José Luis Capelo-Martinez, Jose M. Palomo, Carlos Lodeiro

**Affiliations:** a BIOSCOPE Group, LAQV@REQUIMTE, Chemistry Department, Faculty of Science and Technology, NOVA University Lisbon Caparica Campus Caparica 2829-516 Portugal; b PROTEOMASS Scientific Society, BIOSCOPE GROUP Laboratories Departmental Building, Ground Floor, FCT-UNL Caparica Campus 2829-516 Caparica Portugal; c Instituto de Catálisis y Petroleoquímica (ICP), CSIC Marie Curie 2 Madrid 28049 Spain

## Abstract

This work investigates the potential utilization of Cu(i) as a reducing agent for the transformation of the platinum salt K_2_PtCl_4_, resulting in the production of stable nanoparticles. The synthesized nanoparticles exhibit a bimetallic composition, incorporating copper within their final structure. This approach offers a convenient and accessible methodology for the production of bimetallic nanostructures. The catalytic properties of these novel nanomaterials have been explored in various applications, including their use as artificial metalloenzymes and in the degradation of dyes. The findings underscore the significant potential of Cu(i)-mediated reduction in the development of functional nanomaterials with diverse catalytic applications.

## Abstract 2

This work investigates the potential utilization of Cu(i) as a reducing agent for the transformation of the platinum salt K_2_PtCl_4_, resulting in the production of stable nanoparticles.

## Graphical abstract

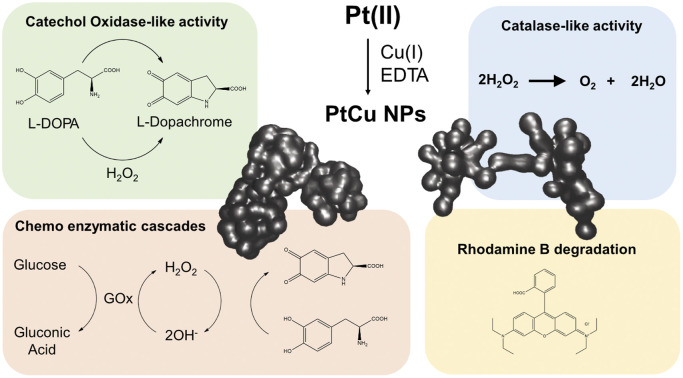

## Introduction

Noble metals such as platinum (Pt) exhibit excellent catalytic properties, for instance, in oxidation catalysis, which is a significant component in the automotive sector,^1^ or hydrogenation catalysis at the industrial level.^2^ The role of catalysis in modern chemical-based technologies is enormous due to their implication in sustainable chemistry to design chemical products and processes that reduce or eliminate the use and generation of hazardous substances.^3^ The use of noble metals, especially in heterogeneous catalysis, electrocatalysis and photocatalysis applications, is of tremendous importance.^4,5^ However, the main drawbacks are their scarcity in nature and elevated price, although their substitution by other non-noble metals is limited.^6^ To this extent, nanomaterials offer an opportunity to reduce the cost and the size of the materials.^7^ The specific surface area is larger when reducing the size of the material, and therefore it has a more efficient atom-utilization rate.^8^ The interest increased with novel methods in the nanotechnology fields, with the selection of the reducing and stabilizing agents being a key component in the nanomaterial synthesis.^9^

The reducing agents used to obtain the nanomaterials will impact the final result, affecting nucleation and growth of the manufactured nanoparticles, with hydrogen (H_2_), sodium borohydride (NaBH_4_), ethylene glycol, glucose and hydrazine being among the most used.^10^ Among them, the polyol process is one of the most used techniques. Some of the main drawbacks of the polyol process are the use of organic phases or elevated temperatures that can modify the surface capping agents. Some polyols can decompose to give carbon dots at temperatures well below their boiling points.^11^ It is also known that the use of different ligands during the synthesis as well as the capping agents can also impact the nanomaterials structure and alter their catalytic activity and selectivity.^12,13^ The possibility of using a non-noble metal as a reducing agent to produce different platinum, gold, silver and palladium nanoparticles in water media under mild conditions was recently demonstrated. Using Fe(ii) as a reducing agent and polyvinylpyrrolidone (PVP) or polystyrene sulphonate (PSS) as stabilizers, it was possible to produce different noble-metal nanoparticles under seedless^14,15^ or seed-mediated^16^ strategies in which the presence of different ions, like citrate or chloride, plays an important role once they can alter the redox potential of the species, making the reaction more favourable.

This methodology can lead to the combination of different metals in the final material, which can impact their activity due to the strong/optimized electronic and structure effects,^17,18^ as well as with strong resistance to poisonous substances compared with pure Pt nanoparticles.^19^ Different synthetic methodologies are used to produce bimetallic nanoparticles such as seed-mediated,^16^ galvanic replacement^20^ or co-reduction^21^ technology with promising applications in the catalysis area. The interest in copper (Cu) being used in combination with different metals, such as gold, palladium, platinum or silver, has increased in recent years.^22–24^ The presence of copper can decrease the cost of the catalyst and improve the catalytic activity in many reactions, *i.e.* selective hydrogenolysis of biomass-derived glycerol or reduction of 4-nitrophenol by NaBH_4_.^20,25,26^

Considering previous work, we have developed a new one-pot and seedless aqueous-based synthetic route to produce well-dispersed PtCu NPs with two different shapes, mulberry-like clusters and dendritic NPs, using copper(i) as a reducing agent in the presence of ethylenediaminetetraacetic acid (EDTA). This simple Cu(i)/EDTA synthesis can be implemented in the absence or presence of PVP as a stabilizer to manufacture well-dispersed PtCu NPs.

We have carefully investigated the catalytic properties of the PtCu NPs by analyzing aromatic compounds reduction and, particularly, as a new artificial metalloenzyme or in a chemoenzymatic cascade in the oxidation of 3,4-dihydroxy-l-phenylalanine (l-DOPA), a catalase-like activity artificial enzyme or as efficient catalysts for rhodamine B degradation.

## Experimental

### Materials

Potassium tetrachloroplatinate (K_2_PtCl_4_), copper(i) bromide (CuBr), polyvinylpyrrolidone (PVP 40K) and acetonitrile (CH_3_CN) were purchased from Sigma-Aldrich. Hydrated ethylenediaminetetraacetic acid tetrasodium salt (EDTA-Na_4_) was purchased from Alfa-Aesar. All reagents were used without further purification. Water was ultra-pure grade (type I) obtained with a Milli-Q Simplicity system.

Catalase from Aspergillus niger (CAT) solution (Catazyme®) and glucose oxidase (Gluzyme® Mono 10.000 BG) (GOx) were purchased from Novozymes (Copenhagen, Denmark). Hydrogen peroxide (33%) and ethyl acetate were from Panreac (Barcelona, Spain). 3,4-Dihydroxy-l-phenylalanine (l-DOPA) was from Alfa Aesar (Massachusetts, EEUU). Sodium phosphate, sodium bicarbonate, sodium acetate, sodium borohydride, dioxane, and rhodamine B (RhB) were from Sigma-Aldrich (St. Louis, MO, USA). *n*-Hexane (98%) and acetonitrile (ACN) were from Scharlau (Madrid, Spain).

### Methods

#### Nanoparticle synthesis

Pt NPs were produced through the reduction of K_2_PtCl_4_ applying CuBr assisted by ethylenediaminetetraacetic acid sodium salt (EDTA) with/without the addition of PVP in a mixture of aqueous : acetonitrile solution (5% (v/v) CH_3_CN in H_2_O).

Briefly, for PtCu1, a round-bottom flask with 15 mL of ultrapure water was immersed in a thermostatic oil bath at 60 °C. With the temperature stabilized and under vigorous magnetic stirring, 2 mL of an aqueous solution containing 10 mmol of K_2_PtCl_4_ were added. Then, 1 mL of CH_3_CN solution containing 0.12 M CuBr freshly prepared was injected, followed by a fast addition of 2 mL of an aqueous solution of 0.1 M EDTA. The reaction was allowed to elapse for 120 min, and then the solution was cooled to room temperature (RT). After this time, the reaction mixture was centrifuged at 13 000 rpm for 30 minutes (3 times) and resuspended in an aqueous solution of 1.5 mM EDTA to a final volume of 12 mL.

For the synthesis in the presence of PVP (PtCu2), a round-bottom flask with 13 mL of ultrapure water was immersed in a thermostatic oil bath at 60 °C. With the temperature stabilized and under vigorous magnetic stirring, 2 mL of an aqueous solution containing 10 mmol of K_2_PtCl_4_ and 2 mL of 0.1 M PVP were added. Then, 1 mL of CH_3_CN solution containing 0.12 M CuBr freshly prepared was injected, followed by the addition of 2 mL of an aqueous solution of 0.1 M EDTA. The reaction was allowed to elapse for 120 min, and then the solution was cooled to room temperature (RT). After this time, the reaction was centrifuged at 13 000 rpm for 45 minutes 2 times, resuspending in an aqueous solution of 1.5 mM EDTA, and one additional cycle resuspending in ultrapure water to a final volume of 12 mL.

#### Catechol oxidase-like activity assay

3,4-Dihydroxy-l-phenylalanine (l-DOPA) (4 mg, 1 mM) was added to 20 mL of 100 mM buffer sodium phosphate pH 7, 40 : 60 ACN : H_2_O and 30 : 70 dioxane : H_2_O. To initiate the reaction, 10 μL of Pt catalyst was added to 3 mL of DOPA solution, and the mixture was slightly stirred (roller) at room temperature or 50 °C. In the case of Pt catalysts, at different times the mixture was measured at 475 nm in a JASCO V-730 UV-spectrophotometer. Then, the Abs per min was calculated with these values in each case. In the case of tyrosinase, the absorbance increase was directly measured at 475 nm on the UV spectrophotometer using the kinetic program. The enzyme activity unit (U) was defined as the amount of enzyme causing an increase of absorbance by 0.001 min^−1^ at 25 °C.^27^

#### Catalase-like activity assay

Hydrogen peroxide (H_2_O_2_) (33% (w/w)) solution was prepared to obtain a final concentration of 50 mM, in 25 mM buffer sodium bicarbonate pH 10, 25 mM buffer sodium phosphate pH 8.5 and 7, distilled water and 25 mM buffer sodium acetate pH 4. To start the reaction, 10 μL of the Pt catalyst or 100 μL of Catazyme® 25 L (31 mg mL^−1^) was added to 2 mL or 10 mL respectively of the 50 mM solution at room temperature. The reaction was followed by measuring the degradation of H_2_O_2_, recording the decrease of absorbance spectrophotometrically at 240 nm in quartz cuvettes of 1 cm path length at different times. To determine the catalase activity for each catalyst, the ΔAbs per min value was calculated using the linear portion of the curve (ΔAbsS).^28^

#### Oxidation of l-DOPA in the presence of H_2_O_2_

3,4-Dihydroxy-l-phenylalanine (l-DOPA) (4 mg, 1 mM) was added to 20 mL of 40% acetonitrile. To initiate the reaction, 10 μL of Pt catalysts were added to 3 mL of DOPA solution and 100 mM H_2_O_2_ (33% (w/w)). The mixture was slightly stirred (roller) at room temperature. The mixture was measured at 475 nm at different times in a JASCO V-730 UV-spectrophotometer. Then, the Abs per min was calculated with these values in each case. The enzyme activity unit (U) was defined as the amount of enzyme causing an increase of absorbance by 0.001 min^−1^ at 25 °C.^27^

#### Chemo-enzymatic cascades

##### GOx cascade

3,4-Dihydroxy-l-phenylalanine (l-DOPA) (4 mg, 1 mM) was added to 20 mL of 40% ACN. To initiate the reaction, 0.5 mL of Gluzyme Mono (10.8 mg mL^−1^, 100 mg of solid per mL) was added to 2 mL of l-DOPA solution and 0.5 mL of glucose (1 M). The mixture was slightly stirred (roller) at room temperature. After 5 min, 10 μL of Pt catalysts were added to the mixture. At different times, a sample of the reaction was measured at 475 nm in a JASCO V-730 UV-spectrophotometer. Then, the Abs per min was calculated with these values in each case. The enzyme activity unit (U) was defined as the amount of enzyme causing an increase of absorbance by 0.001 min^−1^ at 25 °C.^27^

#### Catalase cascade

3,4-Dihydroxy-l-phenylalanine (l-DOPA) (4 mg, 1 mM) was added to 20 mL of 40% acetonitrile. To initiate the reaction, 100 μL of a 1 mg mL^−1^ solution of Catazyme® 25 L (31 mg mL^−1^) was added to 2 mL of l-DOPA solution and 100 mM H_2_O_2_ (33% (w/w)). The mixture was slightly stirred (roller) at room temperature. After 15 min, 10 μL of the Pt catalyst was added to the mixture. At different times the mixture with the Pt catalyst was measured at 475 nm in a JASCO V-730 UV-spectrophotometer. Then, the Abs per min was calculated with these values in each case. The enzyme activity unit (U) was defined as the amount of enzyme causing an increase of absorbance by 0.001 min^−1^ at 25 °C.^27^

#### Degradation of rhodamine B

Rhodamine B (1.2 mg, 48 ppm) was added to 25 mL of distilled water or 40 : 60 ACN : H_2_O. To initiate the reaction, 200 μL of Pt catalysts were added to 1 mL or 2.5 mL of rhodamine B solution in the presence of 250 mM H_2_O_2_ (33% (w/w)) or 3 mg NaBH_4_ (1.2 M), respectively. The mixture in all cases was slightly stirred (roller) at room temperature. At different times samples were measured at 400–700 nm in a JASCO V-730 UV-spectrophotometer to determine the rhodamine degradation.

#### Characterization

All NPs have been characterized by all spectroscopy and standard chemical techniques. Dynamic light scattering (DLS) was done in a MALVERN model ZS instrument (PROTEOMASS Scientific Society, BIOSCOPE facility). Ultraviolet-visible (UV-vis) spectroscopy was performed using a Jasco-650 spectrophotometer with controlled temperature (PROTEOMASS Scientific Society, BIOSCOPE facility). The high-resolution transmission electron microscopy (HRTEM) was performed on a JEOL JEM 2100F 80–200 kV microscope and HAADF in a double-corrected FEI Titan G3 Cubed Themis 60–300 kV microscope through the International Iberian Nanotechnology Laboratory (INL) facility. All TEM samples were prepared by placing a drop of the sample onto a TEM copper or gold grid and air-dried (TED-PELLA Co.). The size of particles and dispersion histograms have been calculated from TEM micrographs using the ImageJ (Fiji) package.^29^ Interplanar spacings in the nanostructures were calculated by Fourier transform (FT) using the ImageJ (Fiji) digital micrograph suite.

Inductively Coupled Plasma-Optical Emission Spectrometry (ICP-OES) studies were carried out in an ICPE-9000 Multitype ICP emission spectrometer from Shimadzu equipped with a nebulizing system and using optical emission spectroscopy for detection through the INL facility.

X-ray photoelectron spectroscopy (XPS) was carried out in an ESCALAB250Xi (Thermo Fisher Scientific) through the INL facility. Analyzer: hemispherical analyser; analysis area (field of view on the sample): defined by the X-ray spot size. X-ray source: monochromated Al Kα (*hν* = 1486.68 eV) radiation, operated at 220 W, 14.6 kV, spot size 650 μm. The XPS spectra were collected at pass energies of 100 eV and 40 eV for survey spectra and individual elements respectively. The energy step for individual elements was 0.1 eV. The XPS spectra were peak fitted using Avantage data processing software. For peak fitting, the Shirley-type background subtraction was used. All the XPS peaks are to be referenced to the adventitious carbon C 1s C–C peak at 284.8 eV. Quantification has been done using sensitivity factors provided by the Avantage library. Charge neutralization was achieved with both low-energy electron and argon ion flood guns (0.5 eV, 100 μA and 70 μA current respectively) during XPS measurements. Samples were deposited by multiple cycles of drop casting of the corresponding solutions over 2.5 days on clean Si wafers followed by drying in air. The solutions were sonicated every time before drop casting.

## Results and discussion

Based on the standard redox potentials known for [PtCl_4_]^2−^/Pt^0^ (+0.75 V *vs.* NHE) and Cu(ii)/Cu(i) (+0.159 V *vs.* NHE), the reduction of Pt(ii) mediated by Cu(i) should be a spontaneous process under normal conditions. However, the potential value for the redox couple Cu(ii)/Cu(i) strongly depends on the nature of the ligand and the halogen counterion.^30^ Also, the redox potential and disproportionation of Cu(ii)–Cu(i) is very sensitive to the solvent.^31,32^ Disproportionation is not rapid or extensive under most conditions but rather requires a combination of an appropriate solvent. Cu(i) is less stable to disproportionation in water, and more stable in ACN. It has been stated than an ACN : H_2_O mixture of 6% (v/v) can stabilize the Cu^+^ with respect to Cu^2+^.^33^

The disproportionation of Cu(i) is generally described by eqn (1):

1 2Cu(i)X ⇌ Cu(0) + Cu(ii)X_2_

Lowering the ACN : H_2_O ratio below the stability point, and with the addition of EDTA in a water environment, we have explored the reduction of Pt(ii) to produce stable bimetallic nanoparticles using Cu(i) as a reducing metal and using EDTA as a chelating and stabilizing agent as described by eqn (2):

2 Pt(ii) + 2Cu(i) ⇌ Pt(0) + Cu(EDTA)

At room temperature (22 °C) and without the addition of any chelating reagent, the reduction of platinum(ii) to platinum(0) mediated by copper(i) does not proceed (molar ratio of Pt/Cu = 1/6) (Fig. S1a†). Interestingly, upon the addition of EDTA, a color change from pale yellow to black takes place in less than 1 minute, confirming the formation of metallic structures in solution (molar ratio of Pt/Cu/EDTA = 1/6/10) (Fig. S1b and c†).

After nanoparticle formation, the UV-vis spectra showed a band centered at 732 nm. This can be attributed to the copper(ii)–EDTA complex,^34^ giving the supernatant a blue color (Fig. S1d†). The stability constant of Cu–EDTA^2−^ is five orders of magnitude higher than that of Cu(OH)_2_, making the reaction more favourable.^35^ Furthermore, the excess EDTA used should act as a stabilizing agent, since the product obtained was stable in water without naked-eye aggregation signals. It should be noted that EDTA has been recently shown to be a reducing agent in gold nanoparticle synthesis.^36^ In the present case, EDTA is unable to reduce Pt(ii) to Pt(0) at either 40 or 60 °C in the time scale of the reactions (typically less than 1 min, data not shown). Therefore, in this case its role will be as a stabilizer and as a chelating agent of the Cu(ii) formed during the reaction.

Considering that the formation of Pt NPs can be strongly affected by the reaction temperature,^15^ control experiments were performed at different temperatures (40 and 60 °C) using DLS to analyze the hydrodynamic size of the nanoparticles formed. The DLS showed that the size is affected by an increase of 20 °C due to a faster reduction, consequently giving smaller nanoparticles at the expense of a slightly higher polydispersity index (PDI) (0.12 to 0.16) (Fig. S2a and b†). Furthermore, two different amounts of copper have been analyzed (molar ratio of Pt(ii)/Cu(i) = 1/3 and 1/6, at 60 °C), keeping constant the final amount of EDTA (molar ratio of Pt(ii)/EDTA = 1/10). It was noticed that an increase in Cu evolved into the smallest nanoparticles (Fig. S2c and d†).

**Fig. 1:**
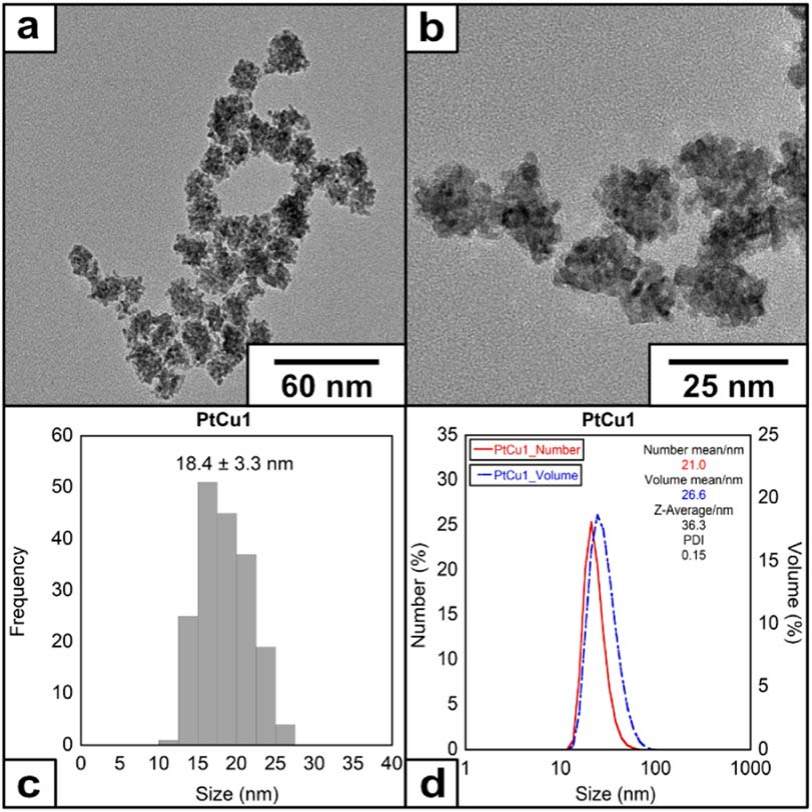
(a) and (b) HRTEM images of the nanoparticles under molar ratio of Pt/Cu/EDTA = 1/6/10 at 60 °C, (c) histogram and (d) DLS of the nanoparticles.

We have selected the molar ratio of Pt/Cu/EDTA = 1/6/10 with the total concentration of K_2_PtCl_4_ of 1 mM at 60 °C to explore the morphology and structure of the metallic materials obtained (denoted as PtCu1). After adding EDTA solution over the Pt(ii)/Cu(i) at 60 °C, a color change from yellow to intense black was observed during the first 10 seconds of the reaction. HRTEM analysis showed the presence of mulberry-like nanoparticles with an average size of 18.4 ± 3.3 nm (Fig. 1) and a ζ-potential of −20.6 mV (Fig. S3†).

**Fig. 2:**
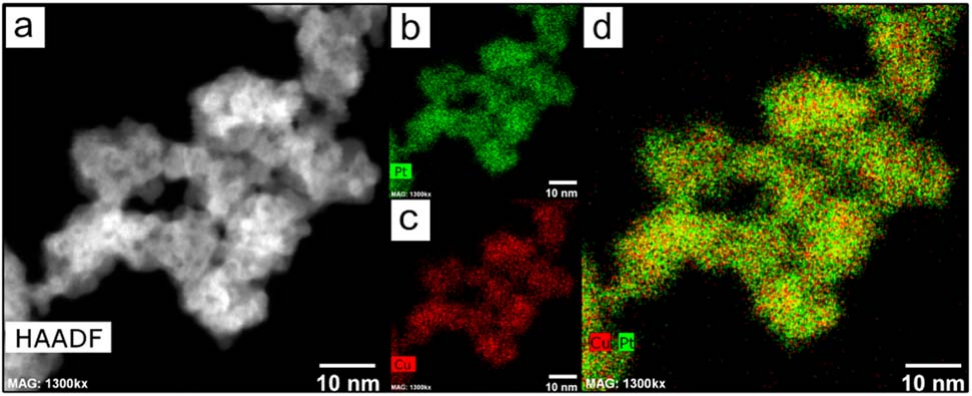
(a) HAADF-STEM image and (b–d) EDX mapping images of the PtCu1 nanoparticles.

The nanoparticles were analyzed through HAADF-STEM to investigate the metallic composition. Fig. 2 shows that the NPs were formed by the combination of Pt and Cu (Fig. 2). The line scan and EDS analysis confirmed the homogeneous distribution of both metals through all the nanoparticles (Fig. S4†). ICP analysis confirmed the bimetallic composition (Table S1†) and with a proportion of Pt : Cu of 2.88 : 1.

**Fig. 3:**
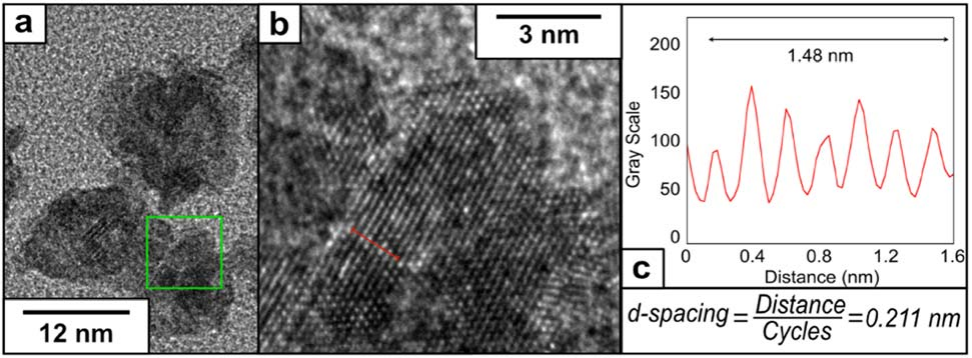
(a) HRTEM image at higher magnification of PtCu1, (b) representative lattice fringe under higher magnification and (c) determination of the inter-planar distance using a plot profile.

Aberration-corrected HRTEM images at higher magnification were recorded to investigate the crystal structure of the NPs. We detected regular lattice fringes in the HRTEM images with regular inter-planar spacings of 0.211 nm (Fig. 3) and 0.205 nm (Fig. S5†), which are close to the inter-planar distance of the PtCu (111) planes and Cu (111) planes reported in the literature.^37,38^ The Fast Fourier Transform (FFT) of the HRTEM confirmed the polycrystalline nature of the PtCu alloy nanoparticles. We have obtained spots at 0.22, 0.20, and 0.13 nm that can correspond to the presence of the Pt (111), Cu (111) and PtCu alloy (200) crystalline planes, confirming the metallic character of our catalyst^37,39^ (Fig. S6†).

To investigate the oxidation states of the metals, the samples were subjected to XPS analysis (Fig. S7†). The expected presence of both metals in different oxidation states was confirmed. The XPS of Pt 4f indicated the presence of Pt(0) and Pt(ii) (71.2 eV to 76.8 eV)^40^ in the final structure. It is worth noticing that in that region also lies the Cu 3p signals which could indicate the presence of Cu(ii) instead of Pt(iv) (76–78.6 eV).^41^ The spectra of Cu 2p showed the peaks that can be attributed to Cu(0) or Cu(i) (932.3 eV) and Cu(ii) (933.9 eV).^42,43^ The disproportionation of Cu may be the reason for the presence of Cu(0) in the final structure, and the presence of oxidation states of Cu can be attributed to the oxidation of surface Cu atoms in air. The ratio of the Pt : Cu through XPS analysis was 1 : 2.25, indicating a surface rich in copper.

**Fig. 4:**
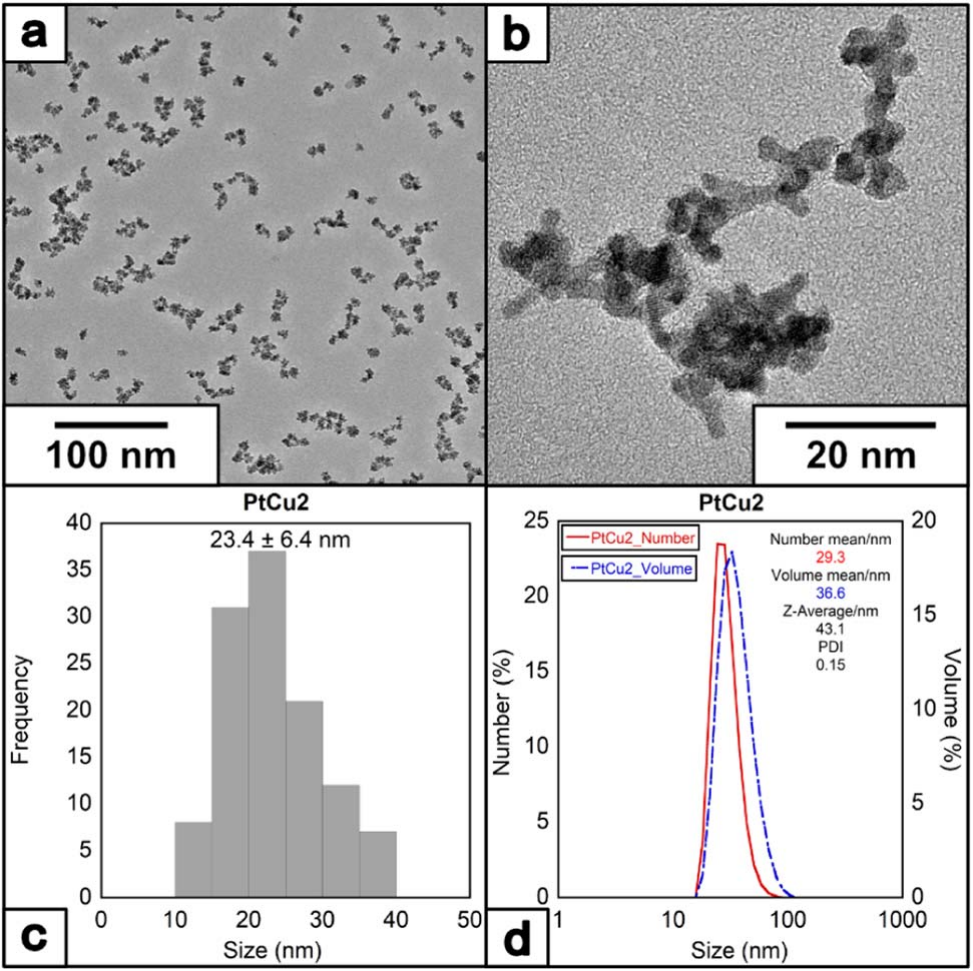
(a) and (b) HRTEM images of the nanoparticles under molar ratio of Pt/Cu/EDTA/PVP = 1/6/10/10 at 60 °C, (c) histogram and (D) DLS of the nanoparticles.

To further investigate the effect of additional stabilizer molecules during the nanoparticle growth, the water-soluble surfactant PVP has been selected to control metal particle formation and deposition rates of metal particles,^44,45^ having been widely used in synthesizing Pt NPs with catalytic or biomedical properties.^46,47^ When the reaction was completed (denoted as PtCu2) in the presence of PVP40K (molar ratio of Pt(ii)/Cu(i)/EDTA/PVP = 1/6/10/10 at 60 °C) (note that PVP concentrations have been expressed based on the monomeric unit of PVP), the size of the NPs increased. Interestingly, in the presence of PVP, the morphology of the nanoparticles changes, from round-like nanoparticles to a more dendritic structure (Fig. 4a and b), with an average size of 23.4 ± 6.4 nm (Fig. 4c and d) and with a ζ-potential of −35.5 mV (Fig. S3†).

**Fig. 5:**
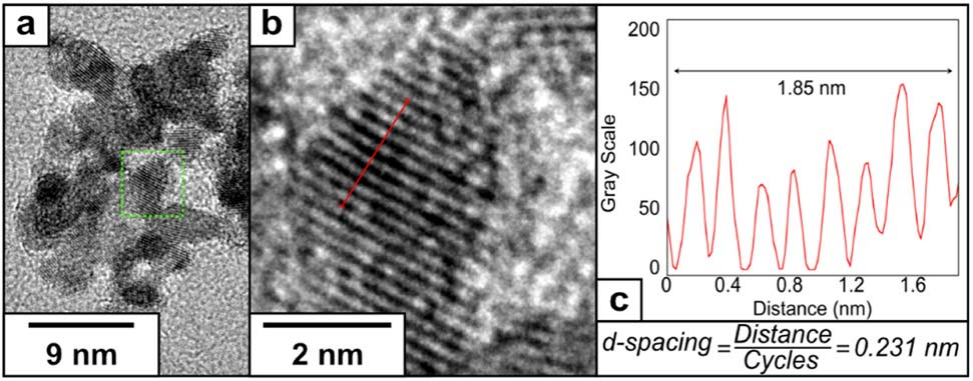
(a) HRTEM image of PtCu2, (b) representative lattice fringe under higher magnification and (c) determination of the inter-planar distance using a plot profile.

The crystal structure of PtCu2 shows a regular inter-planar spacing of 0.231 nm, corresponding to lattice orientation for Pt (111) (Fig. 5). The FFT of the HRTEM images showed the presence of an interplanar distance of 0.20 nm, which can correspond to the presence of Cu (111) (Fig. S8†). The EDS analysis confirmed the presence of platinum and copper in the final nanoparticle (Fig. S9†), with the proportion of Pt : Cu = 2.3 : 1 based on ICP analysis (Table S1†).

The XPS of PtCu2 (Fig. S10†) for Pt 4f showed the presence of Pt(0) (71.5–74.7 eV) and Pt(ii) (72.7–76.0 eV) in the final structure, as well as Cu(0) (74.5–77.6 eV, which was caused by Cu 3p peaks).^40^ In the XPS of the Cu 2p region, fitting the peaks in the range of ∼930–935 eV reveals metallic Cu(0) or Cu(i) (932.3 eV) and Cu(ii) (933.4–934.4 eV). The satellite structure in the range of 940–945 eV confirms the presence of Cu(ii).^42,43^ As with PtCu1, the presence of oxidation states of Cu can be attributed to the oxidation of surface Cu atoms in air. The ratio of the XPS Pt : Cu is 1 : 0.97, indicating a surface composition where platinum and copper are present in similar quantities.

### Catalysis studies

#### Determination of the catechol oxidase-like activity of the different Pt nanocatalysts

An exciting application of metal conjugates is mimicking biological catalytic activities, such as oxidase mimicry, for example, polyphenol oxidases, tyrosinases or catechol oxidases. However, the great difficulty to obtain highly stable proteins with a high level of expression makes them an excellent example of type enzyme where artificial metalloenzyme can be a challenge. These enzymes can catalyse: (i) the *o*-hydroxylation of monophenols to *o*-diphenols as well as (ii) the oxidation of *o*-diphenols to produce o-quinones. In contrast, and by definition, catechol oxidase can only catalyze the oxidation of *o*-diphenols to their corresponding *o*-quinones. Here, the catechol oxidase-like activity of the different Pt nanostructures was evaluated using l-3,4-dihydroxyphenylalanine (l-DOPA) as substrate. This compound does not absorb in the visible region; however, the oxidation produces a chromogenic product (dopachrome) which is brown in color and absorbs at 475 nm.

**Fig. 6:**
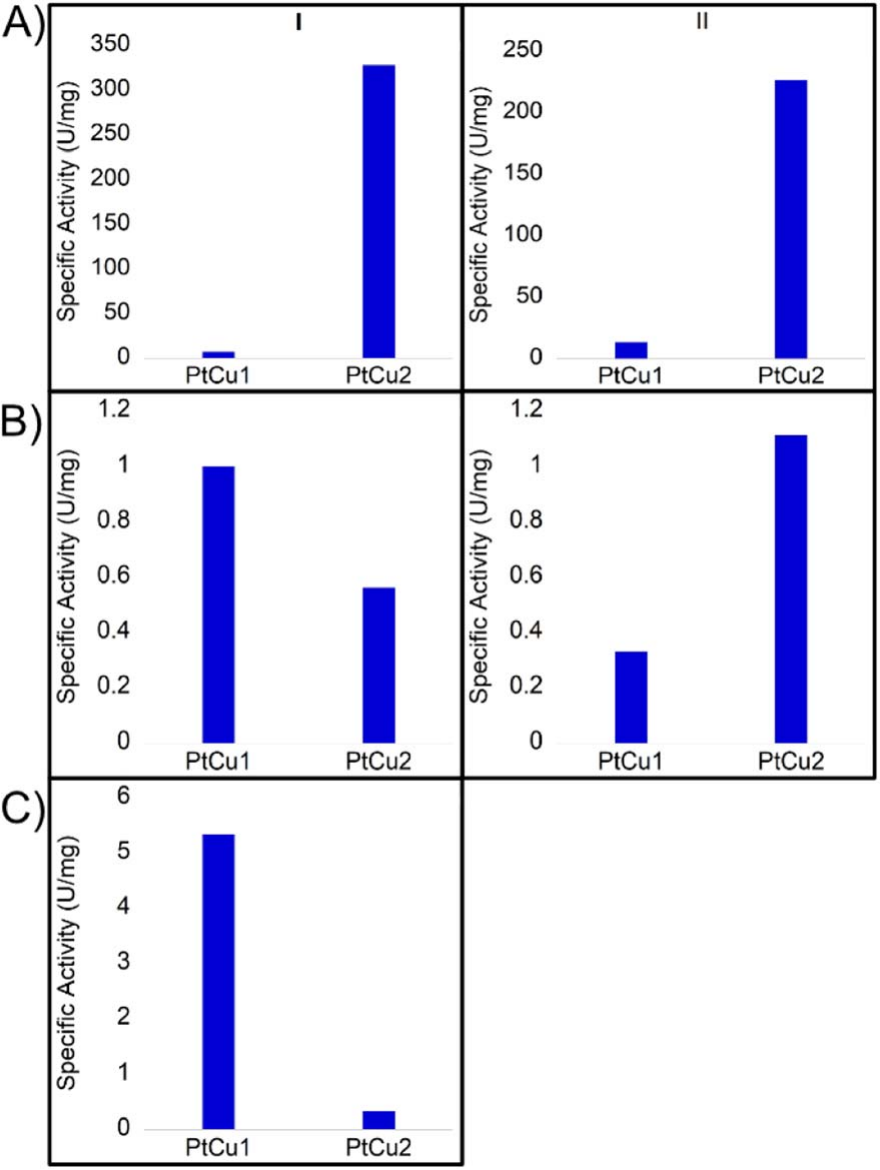
Catechol oxidase-like activity of different Pt catalysts by oxidation of l-DOPA under different conditions at room temperature expressed in values of specific activity (U per mg Pt). (I) room temperature, (II) 50 °C. (A) Buffer sodium phosphate pH 7; (B) 40 : 60 ACN : H_2_O; (C) 30 : 70 dioxane : H_2_O.

The catechol oxidase activity was determined in three different media (buffer solution pH 7, 40 : 60 ACN : H_2_O (v/v) and 30 : 70 dioxane : H_2_O (v/v)) (Fig. 6). At pH 7 and room temperature, PtCu2 showed the highest catechol oxidase activity, with around 330 U per mg with a great difference compared to the PtCu1 (Fig. 6A(I)). Interestingly, the activity seemed extremely affected due to the reaction medium. On this subject, in 40% (v/v) ACN and in 30% (v/v) dioxane all the catalysts showed lower activities compared to pH 7 (Fig. 6B(I) and 6C(I)). We highlight this fact in PtCu2, which showed almost no activity in the presence of 30% dioxane and PtCu1 showed *ca.* 5 times more activity than PtCu2 under 40% ACN conditions.

Another effect studied was the temperature in the case of pH 7 and 40% (v/v) ACN. At 50 °C and pH 7, the PtCu1 catalyst improved the activity approximately two times and PtCu2 showed an activity of 225 U per mg instead of 325 U per mg at rt (Fig. 6A(II)). However, in the presence of 40% ACN, PtCu2 doubled its activity compared to that achieved at room temperature (Fig. 6B(II)).

These differences may be due to the fact that the Pt nanoparticles are coordinated with different ligands. In the case of PtCu2 it is a polymeric ligand (PVP) that is more resistant to temperature, so it could be the reason that the catalytic capacity of the particles has not been affected.

In the case of PtCu1, a buffer solution may destabilize the nanoparticles due to the protonation of the EDTA. Since dioxane can act as a Lewis base, water/dioxane mixtures should allow better dispersion and stabilization of the nanoparticles improving interaction with the system.

### Catalase-like activity

The enzyme catalase is essential for the removal of excess cytoplasmic hydrogen peroxides by converting them to water and molecular oxygen. This enzyme is known to catalyze oxidative and decomposition reactions under very mild and favorable biological conditions. However, the widespread application of this enzyme is restricted by its intrinsic properties, such as denaturation under extreme conditions of pH and high temperatures as well as its protease digestibility. Furthermore, high cost and rigorous storage requirements also limit its applications. Therefore, the development of artificial enzyme mimics has been attracting a lot of attention.

**Fig. 7:**
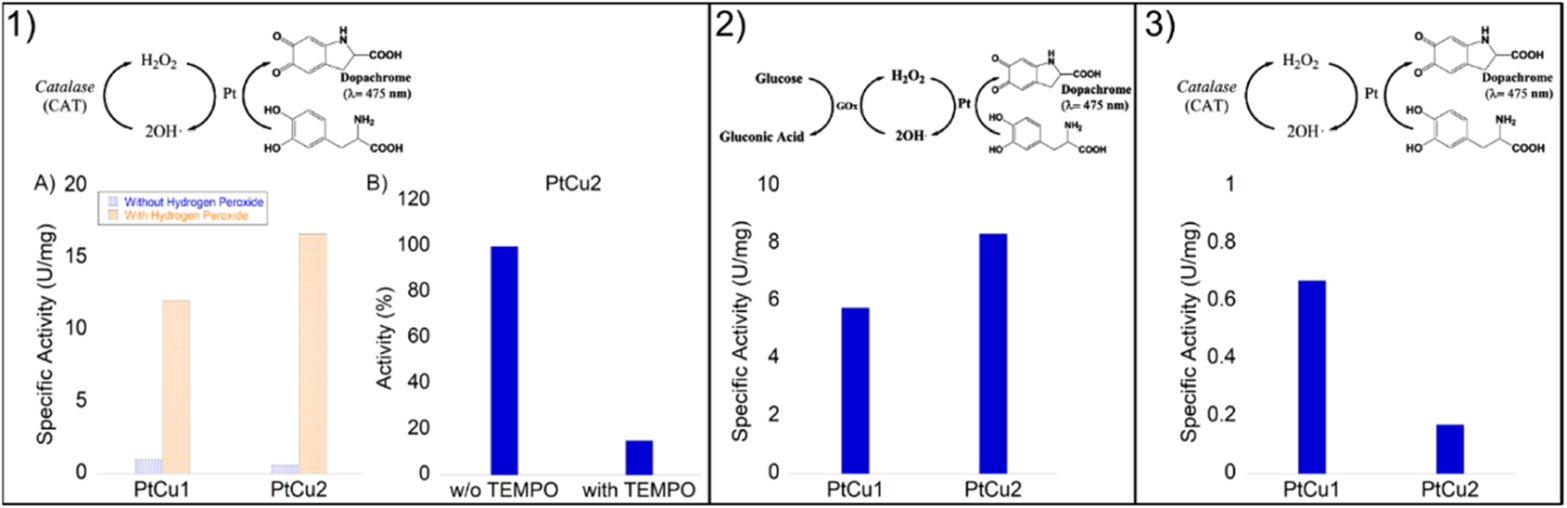
Catalase-like activity of different Pt catalysts under different conditions. All catalysts were evaluated under different conditions (pH 4–10). The best result obtained was for at pH 10 with slightly more than 8 U per mg.

Initially, we tested the peroxidase-like activity of these Pt-catalysts using the glucose assay and no activity was found. However, the degradation of hydrogen peroxide was highly successful (Fig. 7).

In general, the bimetallic catalysts showed higher activity when pH increased (Fig. 7). However, whereas the catalase activity of PtCu1 was barely affected by the pH, this enzymatic activity of PtCu2 was seriously improved (3 times at pH 10) with respect to that in distilled water (Fig. 7).

The PtCu2 hybrid showed similar activity to the natural enzyme (catalase from *A. niger*)^28^ being an excellent candidate as an artificial metalloenzyme. The role of copper in terms of catalytic efficiency depended on the type of reaction where it was used. While in the catechol reaction the high efficiency of Pt in this process^15^ (ref. 15 in the main text) has been previously demonstrated, in the catalase-like activity the efficiency of copper has been demonstrated, which are also much higher than those of other precious metals like Pd or Ag.^48^ Therefore, we could consider that in this process, the catalytic efficiency could be due to the copper.

### Oxidation of l-DOPA in the presence of H_2_O_2_

Recent studies have shown that the presence of hydrogen peroxide in the medium improves the catalytic efficiency of catecholase in the l-DOPA reaction.^28^

**Fig. 8:**
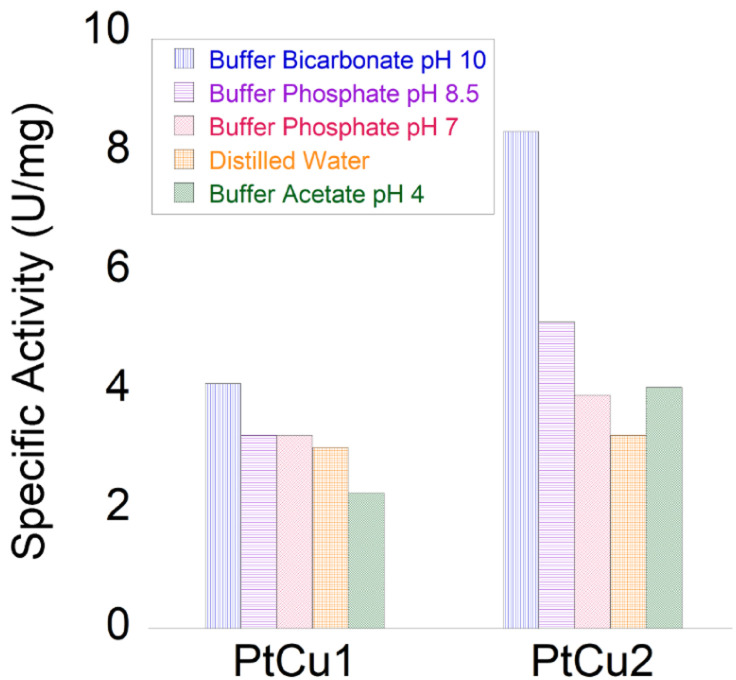
Panel (1) Catechol oxidase-like activity of different Pt catalysts. (1A) Specific activity in the presence of H_2_O_2_ (orange) or the absence of hydrogen peroxide (blue). (1B) Reaction catalysed by PtCu2 with or without TEMPO. Panel (2) Glucose oxidase cascade with a combination of Pt catalysts. Panel (3) Catalase cascade with a combination of Pt catalysts.

Thus, the catecholase activity of Pt catalysts was evaluated in the presence of hydrogen peroxide. Remarkably, in the presence of H_2_O_2_, we noted a catalytic improvement between 10 and 20 times in both nanoparticles (Fig. 8(1A)).

These reactions were carried out under conditions where the Pt catalysts did not present catalase activity, that was at 40 : 60 ACN : H_2_O.

In order to demonstrate that the reaction mechanism goes through a Fenton process, *via* the formation of OH˙ radicals, the reaction using PtCu2 was also carried out in the presence of TEMPO, as a radical scavenger (Fig. 8(1B)). Under these conditions, only 15% activity was observed after 5 min (100% without adding TEMPO) with a clear decrease in the reaction process.

### Chemo-enzymatic cascades

Cascade reactions have been described as efficient and universal tools and are of substantial interest in synthetic organic chemistry. These processes present advantages when compared to the typical single reaction, such as atom economy, step-saving, and therefore high yield and efficiency of the chemical process.^49–51^

Here, the combination of Pt catalysts with enzymes was investigated in order to perform the l-DOPA oxidation by a cascade process. Two different systems were evaluated (Fig. 8, panels 2 and 3). One was based on the *in situ* production of H_2_O_2_ through the oxidation of glucose in gluconic acid due to glucose oxidase (GOx), and another was the *in situ* oxygen production in the media by the degradation of H_2_O_2_ catalyzed by catalase (CAT).

First, a biocatalytic-metal cascade (GOx-Pt nanoparticles) was performed (Fig. 8, panel 2). Both catalysts were added to the water solution containing glucose and l-DOPA as substrates. GOx oxidizes glucose to gluconic acid producing hydrogen peroxide. In this step, the Pt catalyst quickly transforms H_2_O_2_ into OH˙ as we previously demonstrated, which accelerates l-DOPA oxidation to dopachrome.

In this case, PtCu2 was the most active catalyst, with a specific activity of 8.4 U per mg, where PtCu1 showed a specific activity of 6 U per mg (Fig. 8 panel 2). In this cascade, the hydrogen peroxide generated by the bioenzymatic step of GOx corresponded to 50 mM.

A second cascade system is based on the typical acceleration of DOPA oxidation by natural enzymes in the presence of oxygen. To obtain oxygen *in situ* in the reaction, catalase was used to reduce hydrogen peroxide to water and oxygen. Then, this enzyme combined with Pt nanoparticles made the cascade where oxygen biocatalytically produced was used for Pt in the selective oxidation of l-DOPA.

In this case, the reaction was less effective than the previous cascade (Fig. 8 panel 3). This may be due to the fact that Pt has bettr coordination with OH˙ radicals than with O_2_, its oxidative capacity being greater. Although both catalysts showed moderate activities, the PtCu1 catalyst showed 4-times higher activity than PtCu2.

### Degradation of rhodamine B (RhB)

Among the many organic compounds found in wastewater, pollution caused by dyes has been a serious environmental problem for years. Within the general category of colorants, rhodamine B (RhB) is one of the most important xanthene colorants due to its good stability. Therefore, the treatment of these compounds is important for protecting water and the environment in general. Platinum-based nanomaterials have recently been investigated to show high catalytic efficiency for the degradation of RhB.^52^

**Fig. 9:**
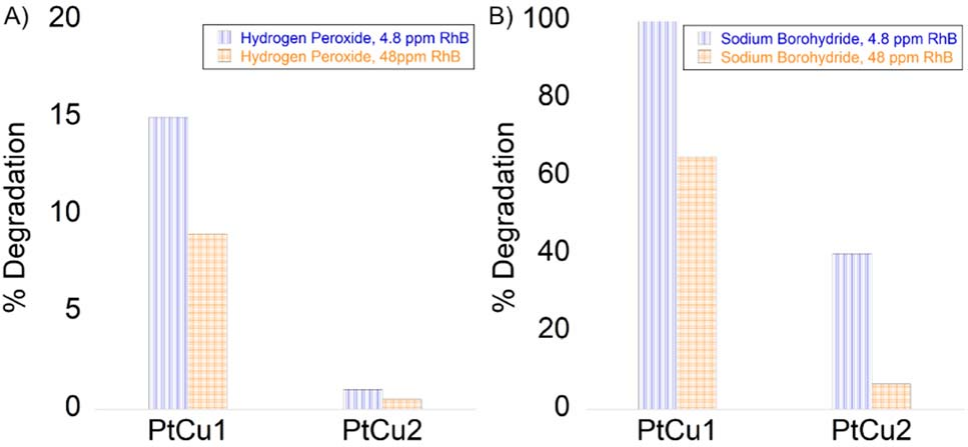
Degradation of rhodamine B (A) in the presence of H_2_O_2_ and (B) in the presence of NaBH_4_. 4.8 ppm of RhB (blue column), 48 ppm of RhB (orange column).

The combination of hydrogen peroxide and platinum has powerful oxidizing properties, as has been proven in the previous experiments described in this work. Hydrogen peroxide reacts with platinum ions to generate active hydroxyl radicals, which responds to RhB degradation. The decrease in RhB was measured by UV-vis absorbance at a wavelength of 550 nm. The reaction was measured at 18 h for 4.8 and 48 ppm, with the best result being PtCu1 degrading around 4 ppm. PtCu2 practically did not degrade the substrate (Fig. 9A).

Another potential use of the Pt-catalysts nanocomposite was explored by catalytic degradation of RhB in the presence of NaBH_4_. The catalytic activity of the nanocomposites was evaluated by varying the concentration of rhodamine B (Fig. 9B). The RhB concentration decreased rapidly immediately after the addition of NaBH_4_ to a concentration of 4.8 ppm catalyzed by PtCu1. PtCu2 only degraded 40% (2 ppm). On the other hand, for 48 ppm at 5 min of reaction, the degradation of RhB was *ca.* 32 ppm for PtCu1, the one that degraded the most rhodamine B.

## Conclusions

We have developed a new synthetic process for bimetallic platinum–copper nanoparticles based on the application of Cu(i) as a reducing agent in the presence of EDTA. This one-pot synthesis allows the obtention of well-dispersed mulberry-like (EDTA stabilized) or dendrite-like (PVP stabilized) nanoparticles with sizes between 15 and 25 nm. The nanomaterials showed the presence of copper in the final structure, making it a fast and straightforward process to obtain bimetallic PtCu nanoparticles. The different nanoparticles were evaluated in a high variety of catalytic reactions. PtCu1 showed the best catalytic performance as an artificial metalloenzyme, with catechol or catalase like-activity, whereas PtCu2 was the most efficient catalyst in the degradation of organic contaminants. Our novel synthesis route introduces a simple and easy-to-modify process for obtaining bimetallic PtCu nanoparticles that present a differentiated catalytic activity according to the presence of bulky (PVP) or discrete (EDTA) stabilizing entities.

## Author contributions

A. F. L., J. F. L., S. N. and C. L. E.: nanomaterials investigation, formal analysis, data curation, methodology and writing the original draft. N. L. G. and J. M. P.: catalysis investigation, formal analysis, data curation, and methodology. A. F. L., J. F. L., C. L., J. L. C., and J. M. P.: supervision, funding acquisition and resources. The manuscript was written through the contributions of all authors. All authors have given approval to the final version of the manuscript.

## Conflicts of interest

There are no conflicts to declare.

## Supplementary Material

NA-005-D3NA00158J-s001
