# Supplementary material for: Copper(i) as a reducing agent for the synthesis of bimetallic PtCu catalytic nanoparticles
Source: Nanoscale Adv. 2023 Jul 20;5(17):4415–23. doi: 10.1039/d3na00158j (PMC10448313; doi:10.1039/d3na00158j)
Supplement: NA-005-D3NA00158J-s001 [file NA-005-D3NA00158J-s001.pdf]

Electronic Supplementary Material (ESI) for Nanoscale Advances.
This journal is © The Royal Society of Chemistry 2023

Copper(I) as reducing agent for the synthesis of bimetallic PtCu catalytic nanoparticles

Adrian Fernández-Lodeiro,[1,2], Javier Fernández-Lodeiro,[1,2], Noelia Losada-Garcia,[3], Silvia Nuti,[1,2], Jose-Luis Capelo-Martinez,[1,2], Jose M.Palomo*[3], Carlos Lodeiro*[1,2]*

[1] BIOSCOPE Group, LAQV@REQUIMTE, Chemistry Department, Faculty of Science and Technology, NOVA University Lisbon, Caparica Campus, Caparica 2829-516, Portugal.

[2] PROTEOMASS Scientific Society, BIOSCOPE GROUP Laboratories, Departamental Building, Ground Floor, FCT-UNL Caparica Campus, 2829-516 Caparica, Portugal

[3] Instituto de Catálisis y Petroleoquímica (ICP), CSIC C/Marie Curie 2, 28049, Madrid, Spain

Table of contents

Figure S1. (a) UV–Vis spectra of the interaction of K_2PtCl_4 with CuBr and EDTA (molar ratio Pt/Cu = 1/6 and Pt/Cu/EDTA= 1/6/10) at 22°C. (b) colour of the Cu+Pt dissolution before adding EDTA, c) colour of the Cu+Pt dissolution after adding EDTA and d) colour of the supernatant Cu(II)-EDTA with the band cantered at 732 nm. 3

Figure S2. (a, b) DLS spectra of the interaction of K_2PtCl_4 with CuBr and EDTA (molar ratio Pt/Cu = 1/6 and Pt/Cu/EDTA= 1/6/10) at 40 ºC and 60 ºC. (c, d) DLS spectra of the interaction of K_2PtCl_4 with CuBr and EDTA (molar ratio Pt/Cu = 1/3 and Pt/Cu = 1/6) at 60 ºC. 4

Figure S3. ζ-Potential of the PtCu1 and PtCu2 in water after the centrifugation cycles. 5

Figure S4. (a-b) line-scanning profiles recorded along the path marked by the yellow line (a) (platinum green line and copper red line) and (c) the EDS spectrum. 5

Figure S5. HRTEM image of PtCu1, (b) representative lattice fringe under higher magnification and (c) determination of the inter-planar distance using plot profile 6

Figure S6. HRTEM of PtCu1 and the corresponding FFT showing spots assigned to Pt (111) (marked as blue), Cu (111) (marked as red) and PtCu (200) (marked as green). 6

Figure S7. PtCu1 High-resolution XPS spectra of a) Pt4f and b) Cu2p 7

Figure S8. HRTEM of PtCu2 and the corresponding FFT showing spots assigned to Pt (111) (marked as blue) and Cu (111) (marked as red). 7

Figure S9. EDS spectra of PtCu2, showing the presence of Pt and Cu in the final nanoparticles. The gold signal is due to the tem grid. 8

Figure S10. PtCu2 High-resolution XPS spectra of a) Pt4f and b) Cu2p. 8

Table S1. ICP results of PtCu1 and PtCu2 nanomaterials 9

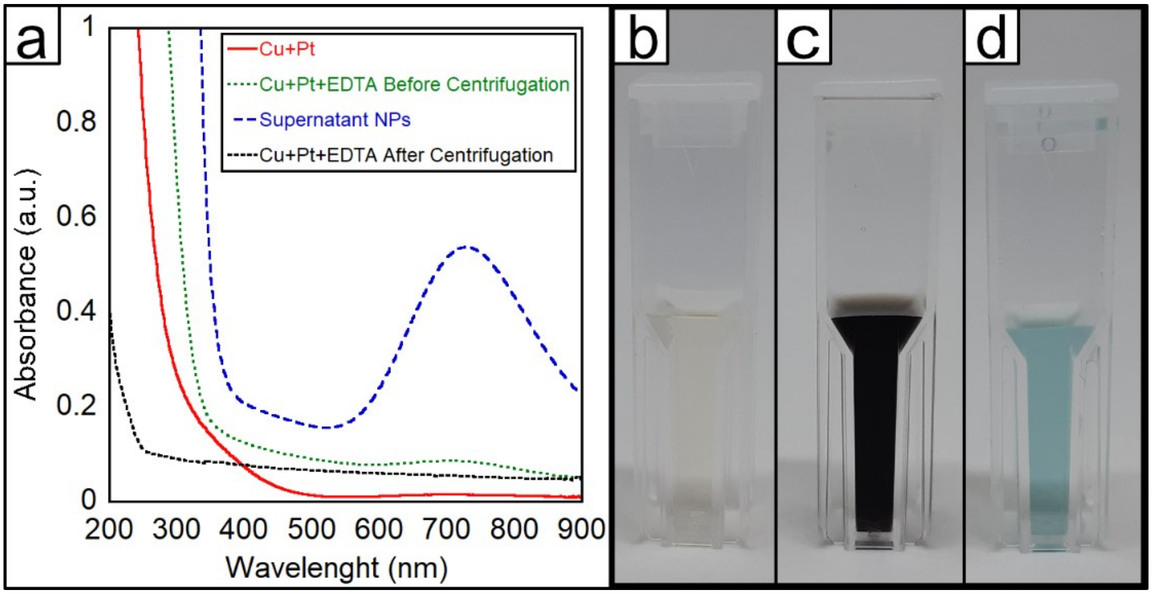

Figure S1. (a) UV–Vis spectra of the interaction of K_2PtCl_4 with CuBr and EDTA (molar ratio Pt/Cu = 1/6 and Pt/Cu/EDTA= 1/6/10) at 22°C. (b) colour of the Cu+Pt dissolution before adding EDTA, c) colour of the Cu+Pt dissolution after adding EDTA and d) colour of the supernatant Cu(II)-EDTA with the band cantered at 732 nm.

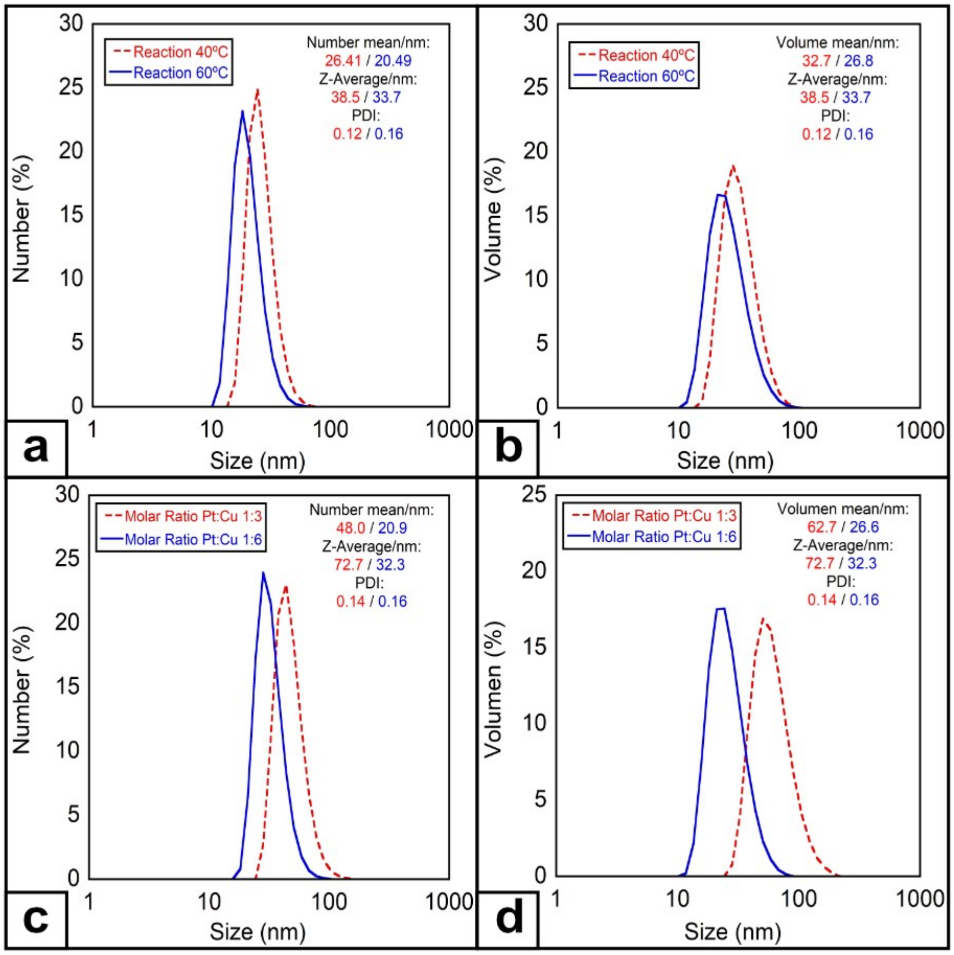

Figure S2. (a, b) DLS spectra of the interaction of K_2PtCl_4 with CuBr and EDTA (molar ratio Pt/Cu = 1/6 and Pt/Cu/EDTA= 1/6/10) at 40 ºC and 60 ºC. (c, d) DLS spectra of the interaction of K_2PtCl_4 with CuBr and EDTA (molar ratio Pt/Cu = 1/3 and Pt/Cu = 1/6) at 60 ºC.

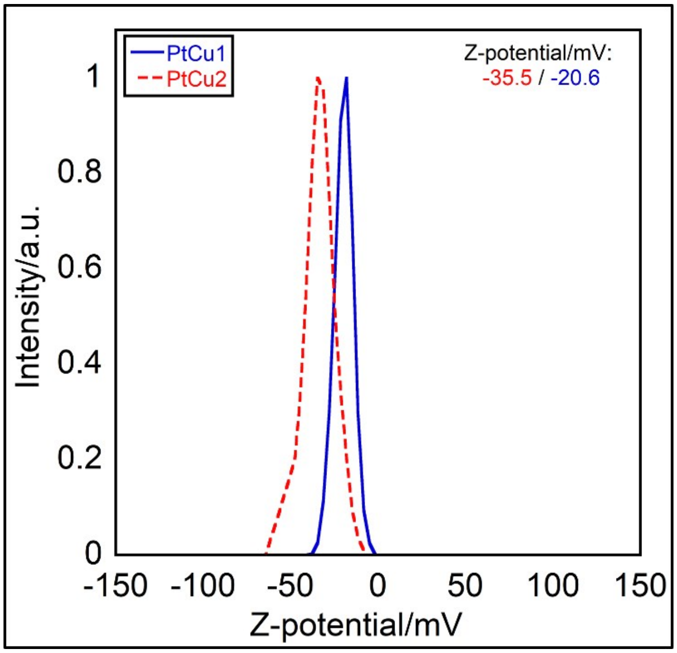

Figure S3. ζ-Potential of the PtCu1 and PtCu2 in water after the centrifugation cycles.

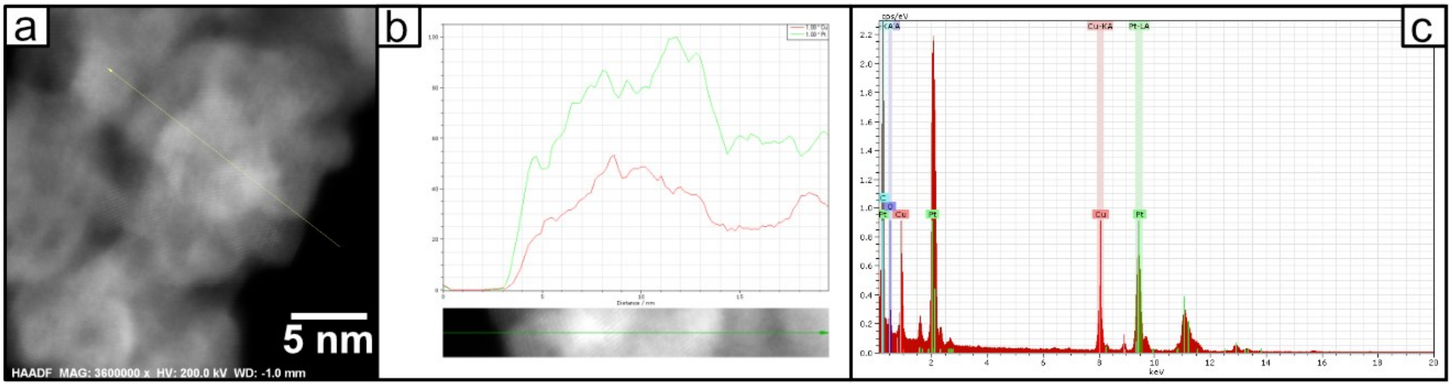

Figure S4. (a-b) line-scanning profiles recorded along the path marked by the yellow line (a) (platinum green line and copper red line) and (c) the EDS spectrum.

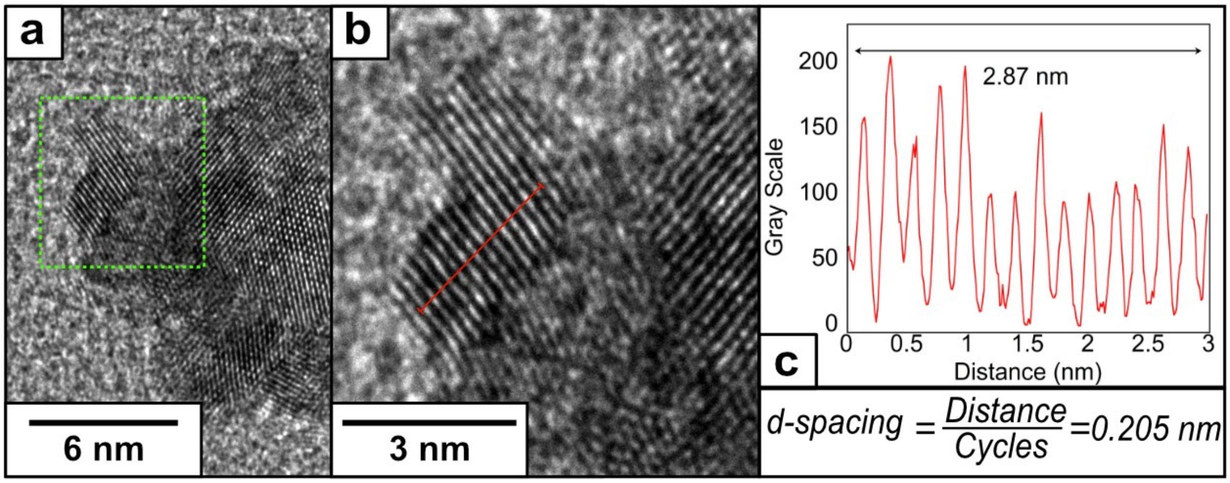

Figure S5. HRTEM image of PtCu1, (b) representative lattice fringe under higher magnification and (c) determination of the inter-planar distance using plot profile.

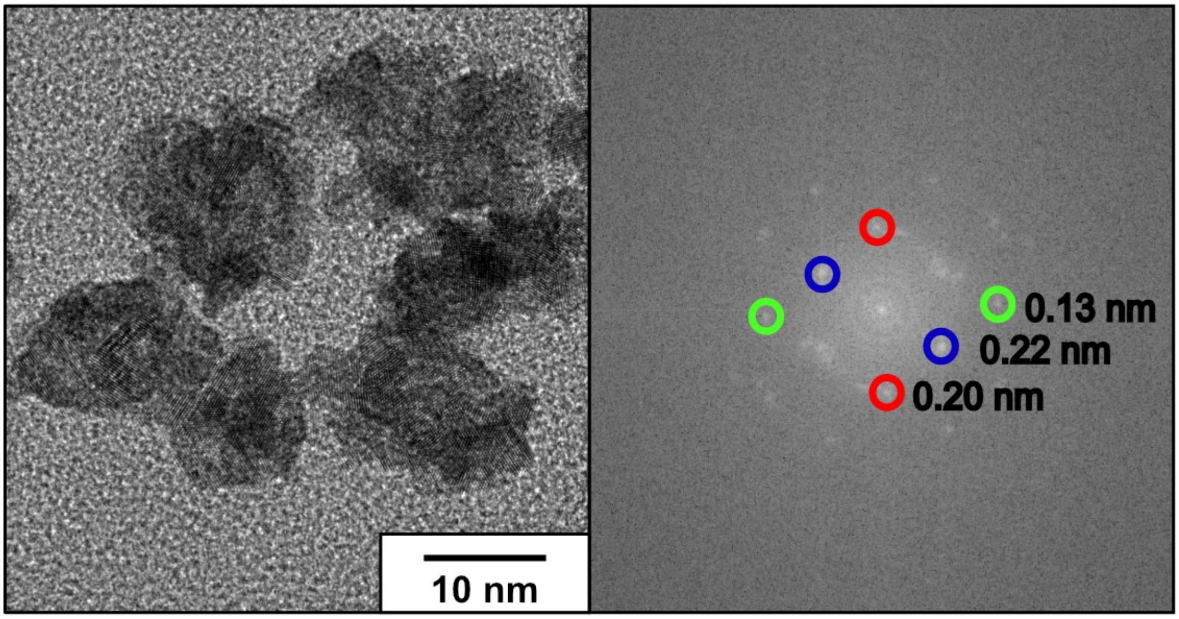

Figure S6. HRTEM of PtCu1 and the corresponding FFT showing spots assigned to Pt (111) (marked as blue), Cu (111) (marked as red) and PtCu (200) (marked as green).

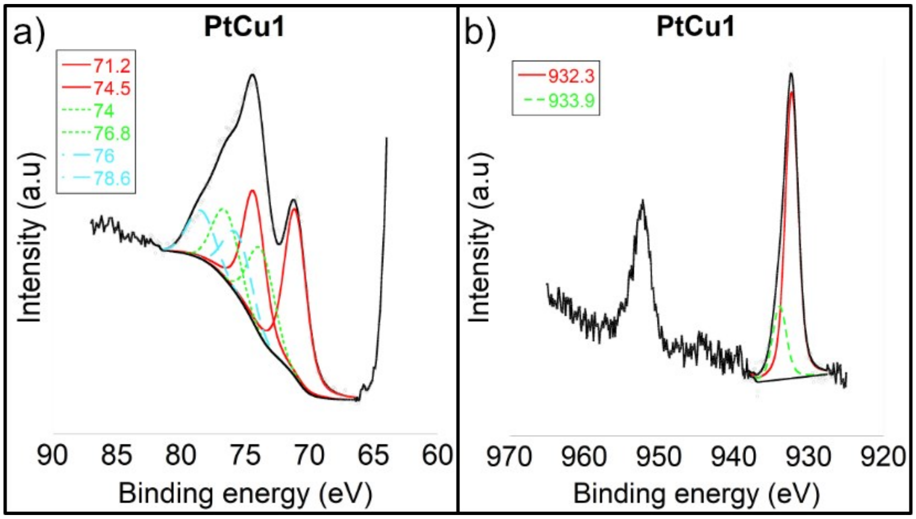

Figure S7. PtCu1 High-resolution XPS spectra of a) Pt4f and b) Cu2p

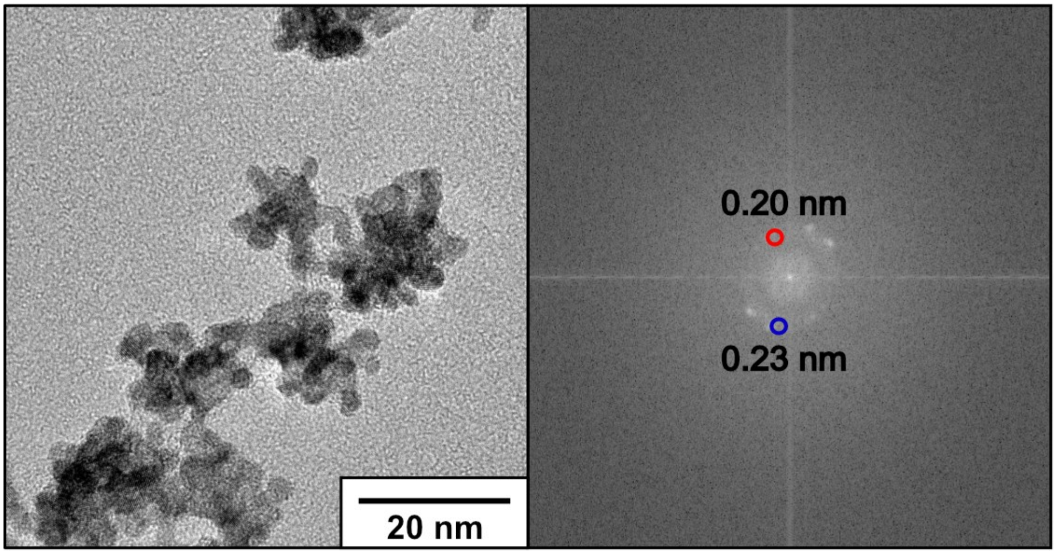

Figure S8. HRTEM of PtCu2 and the corresponding FFT showing spots assigned to Pt (111) (marked as blue) and Cu (111) (marked as red).

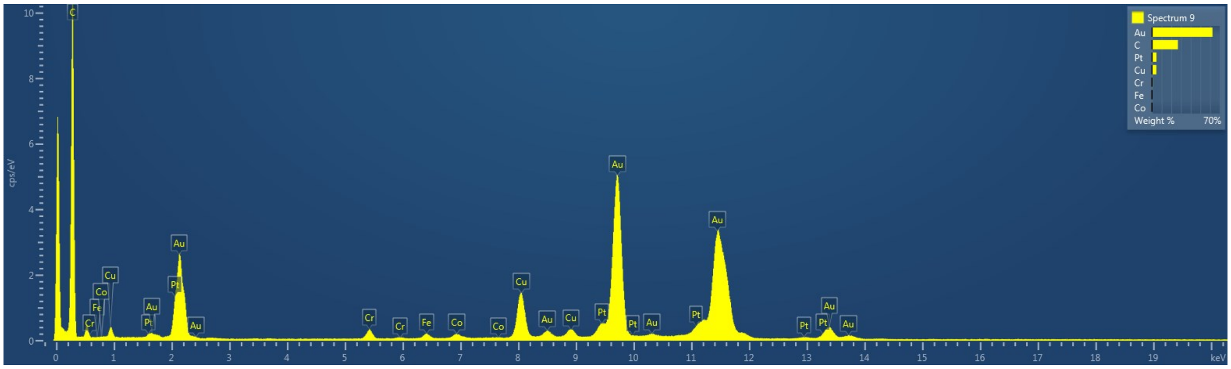

Figure S9. EDS spectra of PtCu2, showing the presence of Pt and Cu in the final nanoparticles. The gold signal is due to the tem grid.

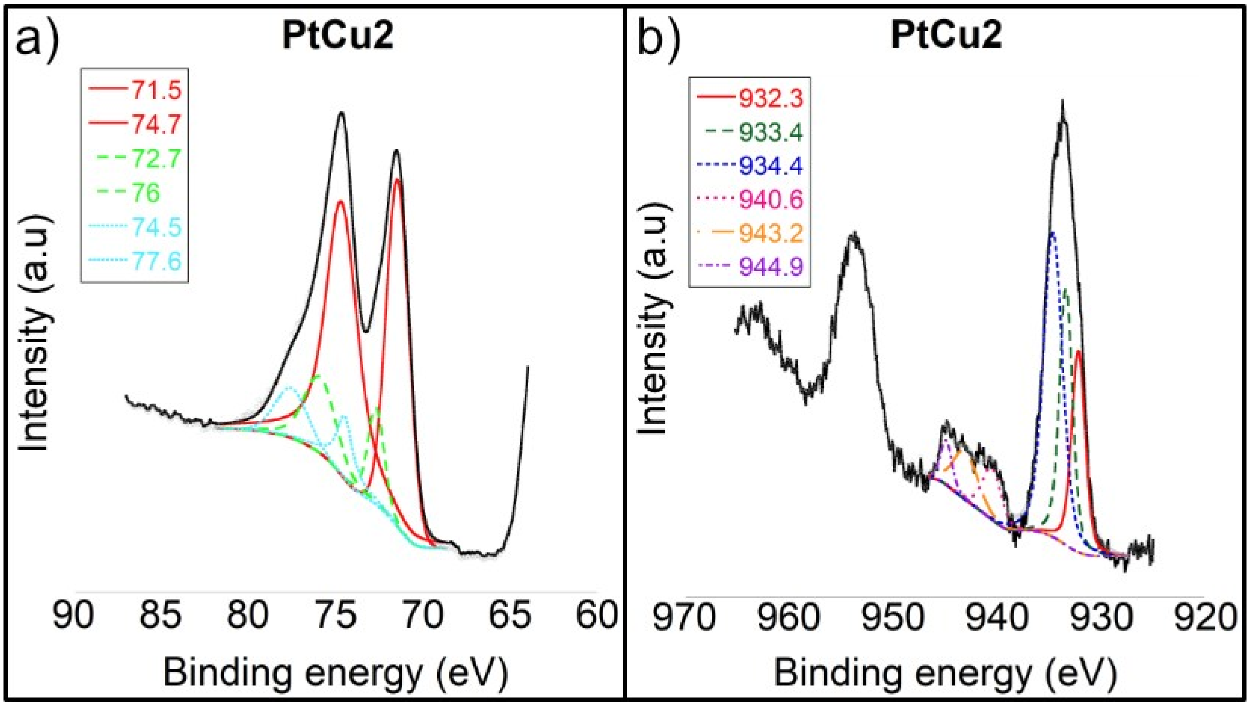

Figure S10.

Figure S10. PtCu2 High-resolution XPS spectra of a) Pt4f and b) Cu2p.

ICP analysis

Digestion procedure

1 mL of sample was added to 50 mL of Mili-Q water. The sample was kept in agitation during 16 h. It was added 1.0 ± 0.1 mL conc. HNO_3 and 0.50 ± 0.05 mL conc. HCl to each sample. The samples were digested for 3 hours 95 ± 5°C. After 3 hours at 95 ± 5°C, the samples were taken from heat source and let cool for at least 30 minutes. The volume of the samples was reconstituted to 50 mL with Mili-Q water.

Table S1. ICP results of PtCu1 and PtCu2 nanomaterials

Sample	Cu [ppm]	Pt [ppm]
**PtCu1**	52	150
**PtCu2**	48	110
